# Letter from Iowa

**Published:** 1882-07

**Authors:** F. W. Goding

**Affiliations:** Tama City, Iowa


### ARTICLE XI.

TAMA CITY, Iowa, July 7, 1882.

EDITORS CHICAGO MEDICAL JOURNAL AND EXAMINER:

The following case, I think, will be interesting to the profession:

June 15, Mrs. B., æt. 28, called at my office and requested my aid in producing an abortion. I refused, and tried to dissuade her from such a course. She stated that she was "' two months along, and if I would not assist her she would do it herself." June 19th a messenger called and stated Mrs. B. was dying.

When I arrived I found the lady in a terrible convulsion—her face fearfully swollen and black; eyes deeply injected and partly open, with the pupils widely and symmetrically dilated. The blood was flowing from her nose and mouth; fingers livid; and each muscle seemed to vie with another in producing the most horrible distortions of face, trunk and extremities. The pulse was rapid, 120, but firm; the surface moist and cool, and she wholly unconscious.

I administered chloroform (Squibbs') and she immediately passed into a semi-conscious state, shortly becoming quite communicative.

She remained in this condition about two hours, when she passed into another convulsion, in which she appeared about as before.

I applied the same means of treatment as before, and soon she was comparatively calm. Noticing the peculiar odor of the eructations, it brought to mind her call at the office. Believing that she had taken some of the many popular remedies to produce abortion, I gave her a hypodermic injection of apomorphia 1-12 grs., which soon produced copious emesis. From the vomited matter I easily obtained the peculiar odor of *oil of tansy*. She admitted taking " over a teaspoonful" of the oil.

Upon examination, I ascertained that she was flowing slightly, and just after I called (two hours later) she passed a two months' fœtus from the vagina. Copious injections of hot water and two drachms of Squibbs' ergot soon checked the flowing, and under the liberal use of stimulants and tonics she has made a rapid recovery.

I call attention to this case as it is the second I have treated here within three weeks, and she states that it is frequently used by the ladies of her acquaintance, she having known of several deaths from its use. Is there no way of preventing the sale of such dangerous drugs to the laity when it is well-known for what purpose they are purchased? F. W. GODING, M.D.

---

It is announced that the French Minister of Marine has placed at the disposition of M. Alphonse Milne Edwards the vessel *Le Travailleur*, for a dredging expedition in the Bay of Biscay, as far as the Canary Islands. The operations, which are to be made with a view to studying the fauna and flora in deep sea, will take place in July and August next.—*British Medical Journal.*

